# Diagnostic Challenges and Surgical Outcomes of Miyazaki Syndrome: A Report of Two Cases and a Systematic Review

**DOI:** 10.7759/cureus.110870

**Published:** 2026-06-15

**Authors:** Filippos Chelmis, Silvia Pecoraro, Dante Magdici, Athanasios Sivridis, Paraskevas Pakataridis, Iliana N Sorotou, Mary Solou, Philip Ho, Athanasios Zisakis, Alexandru Budu

**Affiliations:** 1 Faculty of Medicine, Sofia University "St. Kliment Ohridski", Sofia, BGR; 2 Medicine, Saint James School of Medicine, Chicago, USA; 3 Faculty of Medicine, Medical University of Sofia, Sofia, BGR; 4 Neurosurgery, University General Hospital Attikon, Athens, GRC; 5 Neurosurgery, Queen Elizabeth Hospital Birmingham, Birmingham, GBR

**Keywords:** cerebrospinal fluid overdrainage, cervical epidural venous plexus engorgement, intracranial hypotension, miyazaki syndrome, overshunting-associated myelopathy

## Abstract

Miyazaki syndrome is a rare and underrecognized complication of long-standing cerebrospinal fluid (CSF) diversion. It usually occurs in patients with chronic CSF overdrainage and is caused by enlargement of the cervical epidural venous plexus, which may compress the spinal cord and cause progressive myelopathy or radiculopathy. Because symptoms develop slowly and may resemble degenerative cervical myelopathy or other neurological conditions, diagnosis is often challenging. We report two illustrative cases from our institution. The first patient was a 57-year-old man with a ventriculoperitoneal (VP) shunt placed at birth for neonatal intraventricular hemorrhage (IVH), who developed progressive spastic paraparesis, sensory ataxia, and increasing wheelchair dependence over several years. MRI showed chronic intracranial hypotension with marked cervical venous engorgement and upper cervical cord compression. He underwent shunt revision with the addition of an anti-siphon device, followed by radiological resolution of venous engorgement and partial clinical improvement. The second patient was a 44-year-old woman with congenital hydrocephalus treated with shunting since childhood, who presented with worsening gait disturbance, recurrent falls, impaired hand coordination, orthostatic headaches, and radicular pain. Imaging showed venous congestion at C2 with cord compression and chronic CSF hypotension. Her shunt was revised with placement of a Codman CERTAS® Plus programmable valve and anti-siphon device (Integra LifeSciences, Princeton, NJ, USA), resulting in improved venous drainage, cord decompression, and meaningful clinical recovery. To place these cases in context, we performed a Preferred Reporting Items for Systematic Reviews and Meta-Analyses (PRISMA)-guided systematic review - registered in the International Prospective Register of Systematic Reviews (PROSPERO; CRD420251048122) - of case reports and case series on Miyazaki syndrome or overshunting-associated myelopathy through May 2025 using PubMed, the Cochrane Library, and Google Scholar. Study quality was assessed with Joanna Briggs Institute tools, and data were extracted on patient characteristics, shunt type, valve configuration, anti-siphon device use, symptom latency, imaging findings, treatment, and outcomes. Thirty-one studies, including 37 patients, met the eligibility criteria. The mean age was 43.9 years, and VP shunts were the most common shunt type. The most consistent imaging finding was cervical epidural venous plexus engorgement with cord compression, often associated with pachymeningeal enhancement and slit ventricles. The median interval from the last shunt procedure to symptom onset was 14 years, and valve adjustability did not significantly influence this latency. Most patients were treated by correcting CSF overdrainage through valve pressure adjustment, conversion to a programmable valve, and/or addition of an anti-siphon or gravitational device. All patients treated with an anti-siphon device improved, and earlier treatment was associated with better outcomes. These findings emphasize that Miyazaki syndrome should be considered in shunted patients with progressive myelopathy and imaging signs of intracranial hypotension or CSF overdrainage. Early recognition and correction of siphoning through valve optimization and anti-siphon device placement may reverse venous congestion, improve neurological function, and prevent permanent spinal cord injury.

## Introduction

Historically first described by Miyazaki in 1998 [1], Miyazaki syndrome is a rare medical condition characterized by cervical myelopathy or radiculopathy due to epidural venous congestion secondary to chronic intracranial hypotension, typically caused by cerebrospinal fluid (CSF) overdrainage [2,3]. Although the underlying pathophysiology is not entirely understood, it is based on the Monro-Kellie principle, in which any reduction in CSF volume is compensated by an increase in intracranial blood volume and further affected by the dysfunction of the natural Starling resistor, resulting in engorgement of the venous system due to the absence of valves in the cervical epidural venous network, consequently compressing the spinal cord [2,4,5]. Magnetic resonance imaging (MRI) demonstrates features of CSF hypovolemia, with congestion of the cervical epidural venous plexuses being a key and frequently observed sign. This is often seen in conjunction with other indicators such as diffuse pachymeningeal enhancement and evidence of tonsillar descent [6]. Unlike typical CSF hypotension, which usually manifests with orthostatic headaches, patients with Miyazaki syndrome often experience a gradual onset of progressive motor deficits and pyramidal signs in the absence of, or with minimal characteristic hypotension symptoms [2,6-8]. The rarity of this syndrome has prevented the formulation of standardized treatment protocols, with current management approaches predominantly based on isolated case series and clinical observations [6,9,10]. Our study conducts a comprehensive systematic review on the diagnosis and management of this condition, with case examples from our institution illustrating the diagnostic and clinical challenges associated. Despite more than 25 years since the original description, fewer than 40 cases of Miyazaki syndrome have been reported, and no consensus exists on diagnostic criteria, optimal imaging protocol, or surgical strategy. Patients are frequently misdiagnosed with degenerative cervical myelopathy and undergo decompressive surgery that fails to address the underlying overdrainage and risks epidural bleeding from the engorged venous plexus. To address this gap, we present two illustrative institutional cases, together with the most comprehensive Preferred Reporting Items for Systematic Reviews and Meta-Analyses (PRISMA)-guided systematic review of the syndrome to date, so that the bedside picture and the pooled evidence are available in a single reference [11].

## Case presentation

Case 1

**Figure 1:**
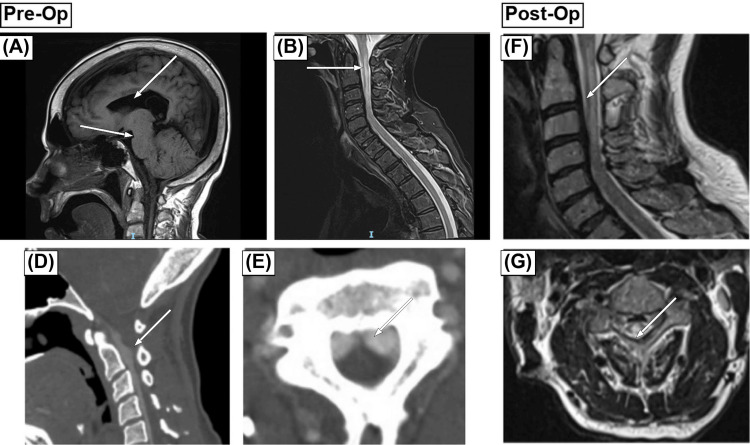
Preoperative and postoperative imaging in Case 1, a 57-year-old man with a VP shunt placed at birth for neonatal IVH. (A) Preoperative sagittal T1 brain MRI showing slit-like lateral ventricles (upper arrow) and brainstem sagging with downward displacement of the midbrain (lower arrow), consistent with chronic CSF overdrainage. (B) Preoperative sagittal T2 cervical MRI demonstrating intramedullary T2 hyperintensity at the cervico-medullary junction extending to C4 (arrow), consistent with myelopathic changes. (C) Preoperative axial T2 cervical MRI at the level of maximal compression showing intramedullary signal change consistent with myelopathy, with circumferential epidural venous plexus engorgement (arrow). (D) Preoperative sagittal CT cervical spine showing the engorged epidural venous plexus (arrow). (E) Preoperative axial CT cervical spine, late post–IV contrast, showing the engorged epidural venous plexus compressing the cord (arrow). (F) Postoperative sagittal T2 cervical MRI after shunt revision with addition of an anti-siphon device, showing resolution of the venous engorgement with persistent intramedullary T2 hyperintensity (arrow) indicating irreversible myelopathic changes due to delayed diagnosis of Miyazaki syndrome. (G) Postoperative axial T2 cervical MRI showing resolution of venous engorgement and restored perimedullary CSF spaces, with persistent intramedullary signal change (arrow) confirming irreversible myelopathy. VP, ventriculoperitoneal; IVH, neonatal intraventricular hemorrhage.

Case 1 illustrates a 57-year-old shunted male patient with a ventriculoperitoneal (VP) shunt placed since birth for neonatal intraventricular hemorrhage, with multiple shunt revisions, the last performed several decades earlier. He presented with progressive spastic paraparesis, sensory ataxia, hand clumsiness, and wheelchair dependence evolving over approximately two decades. He experienced headache episodes, including a nocturnal-onset, non-radiating vertex headache without nausea, photophobia, fever, or neck pain, associated with transient blurred vision that resolved spontaneously; the headache settled after intravenous paracetamol, his Glasgow Coma Scale (GCS) remained 15/15 throughout, and he was discharged. A further symptomatic episode involved bilateral hand numbness and reduced responsiveness without seizure activity, with GCS again 15/15, and was ultimately classified as a headache episode. MRI demonstrated chronic intracranial hypotension, with brainstem sagging and a slit-like third ventricle (Figure 1A), together with longitudinally extensive chronic cervical cord myelopathy from the posterior arch of C1 to the inferior endplate of C4, mild multilevel spondylotic canal narrowing, including thickening/ossification of the posterior longitudinal ligament (OPLL), and ventral epidural venous plexus distension (Figure 1B-1E). Because slit ventricles alone are supportive rather than pathognomonic, the diagnosis was based on the combined clinical and radiological picture. Multidisciplinary review confirmed chronic CSF overdrainage as the underlying cause and ruled out decompressive laminectomy because of the marked venous engorgement. Anti-siphon device insertion was recommended, and the patient consented. The shunt system was revised through a left pectoralis incision, exposing the VP tubing; clear CSF flow was confirmed, and an anti-siphon device (MIETHKE SHUNTASSISTANT® gravitational valve; Christoph Miethke GmbH & Co. KG, Potsdam, Germany) was inserted without intraoperative complications.

Follow-up MRI demonstrated resolution of the cervical venous engorgement and improved peri-medullary CSF spaces (Figure 1F-1G), indicating effective correction of the overdrainage-related intracranial hypotension. Clinically, limb strength was intact and the headache episodes resolved completely; however, persistent myelopathic cord changes and long-standing disability suggested irreversible injury before intervention, with no meaningful subjective improvement in gait and ongoing wheelchair dependence. The modified Japanese Orthopaedic Association (mJOA) score improved from 9 (2+2+2+3) preoperatively to 11 (3+3+2+3) postoperatively, reflecting modest objective lower-limb motor improvement despite limited functional recovery [12]. In activities of daily living, he regained some upper-limb function but continued to require assistance with transfers and remained wheelchair-bound. This case highlights the importance of early recognition and treatment of Miyazaki syndrome secondary to VP shunt overdrainage, as delayed correction may reverse venous engorgement and headache while leaving established myelopathic deficits largely unchanged. He continued physiotherapy, and future posterior decompression was discussed as a potential option in the event of further neurological decline. The patient provided informed consent for publication of this case report.

Case 2

**Figure 2:**
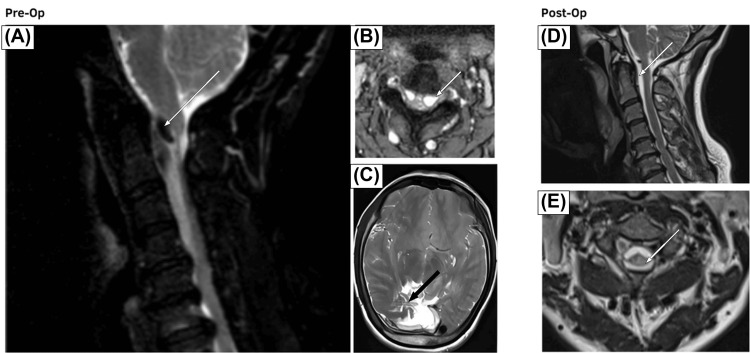
Pre- and postoperative imaging in Case 2 after revision with a programmable CERTAS valve and anti-siphon device. (A) Preoperative sagittal STIR cervical MRI showing the engorged cervical epidural venous plexus compressing the spinal cord (arrow). (B) Preoperative axial gradient-recalled echo (GRE) cervical MRI, late post–IV contrast, showing the engorged epidural venous plexus (arrow). (C) Preoperative axial T2-weighted brain MRI showing slit-like ventricles (black arrow). (D) Postoperative sagittal T2-weighted cervical MRI showing resolution of the venous engorgement with improved CSF spaces (arrow). (E) Postoperative axial T2-weighted cervical MRI showing resolution of the engorgement and cord decompression (arrow). STIR, short tau inversion recovery. CERTAS valve and anti-siphon device (Codman CERTAS® Plus Programmable Valve; Integra LifeSciences, Princeton, NJ, USA).

Case 2 describes a 44-year-old female patient with congenital hydrocephalus, initially treated with a ventriculoatrial (VA) shunt in infancy and later revised to a VP shunt. Her background history included epilepsy treated with carbamazepine and learning difficulties requiring assistance from her sister. Over a seven- to eight-year period, she developed progressive gait instability, recurrent falls, worsening fine motor function, intermittent/orthostatic headaches, and radicular pain affecting both the upper and lower limbs. Neurological assessment demonstrated an unsteady gait, hyperreflexia in the upper and lower limbs, bilateral clonus, upgoing plantars, bilateral Hoffmann’s signs, impaired joint-position and vibration sense in the lower limbs, and a positive Romberg’s test. Imaging demonstrated well-decompressed ventricles with an intact shunt system, features of chronic CSF hypotension, including shallow bilateral subdural collections, smooth dural thickening, brainstem sagging, and pituitary prominence, together with anterior epidural venous engorgement extending from the craniocervical junction to C4 and contributing to cervical canal narrowing and cord compression (Figure 2A-2C). Additional multilevel spondylotic changes were present, including disc-osteophyte bars at C4-C7, but without cord signal change. The case was discussed in the CSF multidisciplinary team meeting, where the findings were considered consistent with shunt overdrainage, with associated cervical venous congestion. Surgical exploration and shunt revision were therefore performed, replacing the old calcified valve with a programmable CERTAS valve and anti-siphon device (Codman CERTAS® Plus Programmable Valve; Integra LifeSciences, Princeton, NJ, USA). Follow-up MRI showed resolution of the venous congestion at the craniovertebral junction and improved venous drainage with cord decompression (Figure 2D-2E). Clinically, follow-up at six and 12 months demonstrated marked improvement in headaches and hand coordination, with an improvement in mJOA score from 14 preoperatively (4+5+2+3) to 17 postoperatively (5+7+2+3) [12].

Both cases illustrate the diagnostic complexity given the pre-existing neurology (linked to the primary cause of hydrocephalus) and underscore the importance of recognizing characteristic imaging features of overdrainage in the presence of a myelopathic deterioration. As with degenerative cervical myelopathy, the postoperative course follows a similar pattern, emphasizing the need for early diagnosis and treatment.

## Discussion

Materials and methods

Search Strategy

A systematic review (and meta-analysis) of the literature was performed according to PRISMA guidelines [12]. The systematic review was registered in the International Prospective Register of Systematic Reviews (PROSPERO; CRD420251048122) [13]. We searched for articles in PubMed/MEDLINE, Google Scholar, and Cochrane libraries for articles related to Miyazaki syndrome. We used the following keywords and combinations (Miyazaki syndrome OR Oversunting-Associated Myelopathy (OSAM) OR OSAM OR Cervical Myelopathy). 

Selection Criteria

After duplicate records were removed, all studies identified through the search were screened by title and abstract based on the predefined inclusion criteria: (1) primary studies, (2) available in full text, and (3) pertained to humans. We excluded all studies (1) not describing overshunting myelopathy, (2) not clearly defining if the myelopathy was due to overshunting or degenerative causes, (3) untranslatable non-English papers, and (4) studies including patients (<16 years).

Study Selection Process

Two reviewers (FC and SP) independently screened the search results by title and abstract. Any discrepancies were discussed with a third author (AB), who acted as an adjudicator, and final decisions were made by consensus. Articles considered potentially eligible underwent full-text assessment to confirm their suitability for inclusion in the review. In addition, the reference lists of all included studies were manually reviewed to identify any relevant articles not captured by the initial search strategy.

Study Appraisal and Quality Assessment

**Figure 3:**
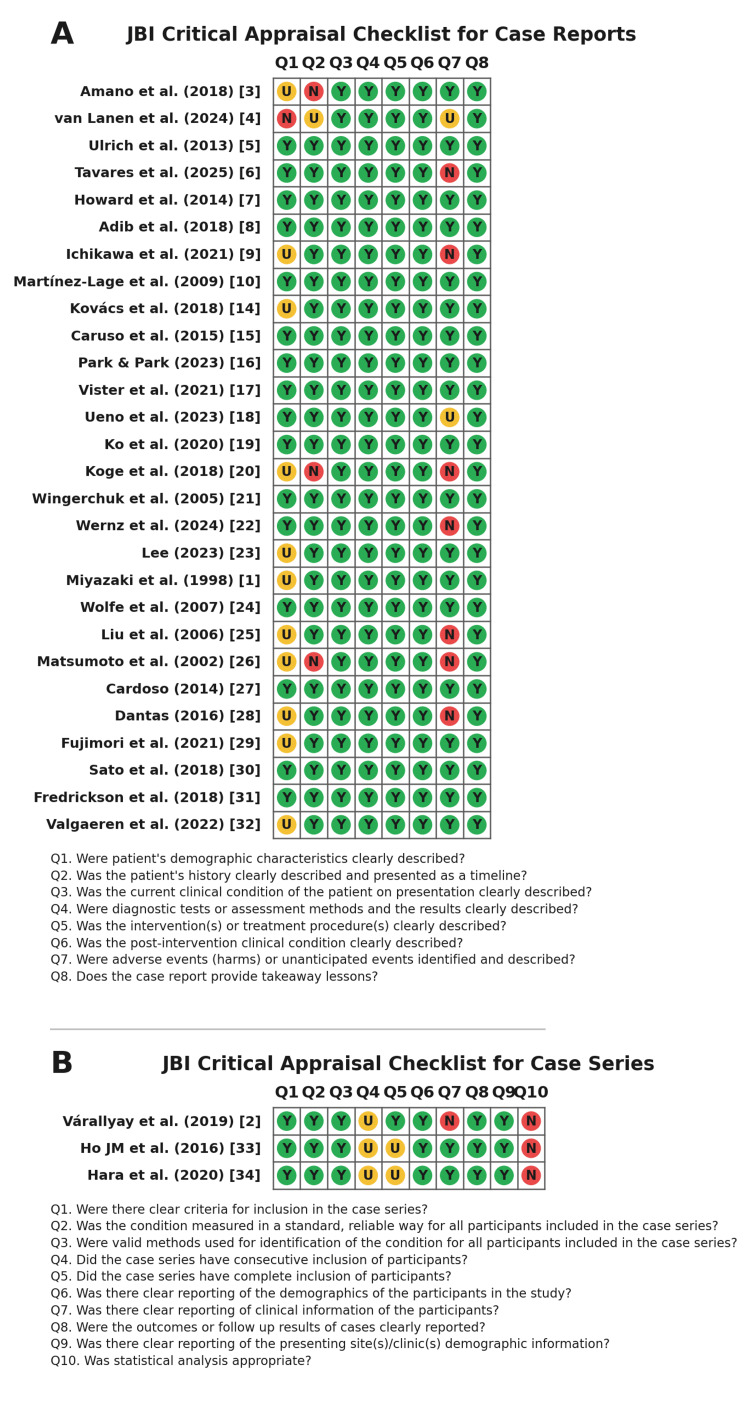
Quality appraisal of included studies using the Joanna Briggs Institute (JBI) critical appraisal checklists. (A) Quality assessment of included case reports using the JBI Critical Appraisal Checklist for Case Reports. (B) Quality assessment of included case series using the JBI Critical Appraisal Checklist for Case Series. Green indicates “yes,” yellow indicates “unclear,” and red indicates “no” for each appraisal item [1-34].

We performed a risk-of-bias assessment using the Joanna Briggs Institute (JBI) critical appraisal tools, tailored to the design of the studies. Due to the scarcity of available literature, only case reports and case series were considered. Accordingly, we applied the JBI checklist specific to each study type, an eight-item checklist for case reports, and a 10-item checklist for case series. Each study was evaluated based on these items, and only those with greater than 70% “yes” responses were included in the review. This threshold ensured that only studies with adequate quality were included, making our findings more reliable (Figure 3) [1-10,14-36].

Data Extraction

For all studies meeting the inclusion and exclusion criteria, a standardized form was utilized to extract and store data from each study in an organized manner. For each included study, we extracted relevant demographic, clinical, radiological, and treatment-related data. These included patient age and sex, indication for shunt placement, shunt and valve type, presence of an anti-siphon device, latency from the most recent shunt implantation to symptom development, duration of cervical symptoms, neurological and clinical manifestations, imaging findings, therapeutic approach, and clinical outcome. Among these, our primary outcomes were valve type, anti-siphon device, time-log of the onset of symptoms after the last shunt implantation, and treatment. All other parameters were considered secondary outcomes. Data extraction was performed independently by two reviewers (FC and IS). Any discrepancies were resolved through discussion, and if consensus could not be reached, a third reviewer acted as arbitrator (AB).

Statistical Analysis

All analyses were performed in Python 3.12.8 (Python Software Foundation, Wilmington, DE, USA) using pandas, NumPy, Matplotlib, and Seaborn, with a two-sided p-value < 0.05 considered statistically significant. The primary analysis evaluated the influence of shunt hardware features, specifically valve type and the presence of an anti-siphon device (ASD), on the time to symptom onset (Time_after_implant) and treatment outcome. The Mann-Whitney U test compared Time_after_implant between adjustable and non-adjustable valves. 

Case Reports and Consent

Two patients from our institution were included, and individual informed consent was obtained in accordance with the Declaration of Helsinki. The case presentation of the two cases was written following the CARE guidelines [37].

Results

**Figure 4:**
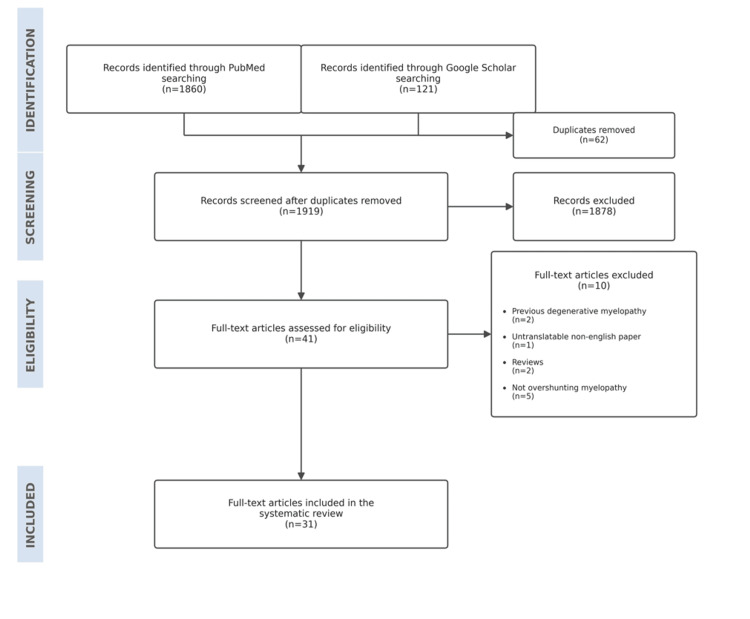
PRISMA flow diagram of the study selection process. PRISMA, Preferred Reporting Items for Systematic Reviews and Meta-Analyses.

The literature screening and study selection process is summarized in the PRISMA flow diagram (Figure 4) [11]. Through the database searches, according to the established search strategy, a total of 1981 articles were selected for subsequent filtering. Of these, 62 duplicate articles were excluded. After screening titles and abstracts, 1,925 records were deemed unrelated to the review question and were excluded. Full-text assessment was subsequently performed for 41 articles; 31 studies met the eligibility criteria and included a total of 37 reported patients (Figure 4). We also included two additional patients from our institution (not included in the statistics).

The cohort included 18 females and 19 males with a mean age of 43.9 years and a median of 41 years (age range at symptom onset: 16 - 83 years). The underlying reasons for initial shunt placement were diverse. The most frequent broad category was hemorrhage-related hydrocephalus (N=10, 27.0%). This included hydrocephalus secondary to subarachnoid hemorrhage (SAH) (N=5), intraventricular hemorrhage IVH (N=2), normal pressure hydrocephalus (NPH) following SAH (N=2), and one case involving significant bilateral chronic subdural hematomas associated with an arachnoid cyst (N=1). The second most common indication was cyst-related conditions, which accounted for nine cases (24.3% of the total cohort). This category was primarily composed of arachnoid cysts (N=7, 18.9%), with individual cases of a large porencephalic cyst (N=1, 2.7%) and a colloid cyst (N=1, 2.7%) also reported. The next indication was congenital hydrocephalus with N=6 cases reported (16.2%). Then, aqueductal stenosis with N=5 cases (13.5%). Tumor-related hydrocephalus was found in N=3 cases (8.1%, pilocystic astrocytoma N=2 and meningioma N=1). Idiopathic hydrocephalus (N=2, 5.4%) and post-infectious (meningitis) hydrocephalus (N=1, 2.7%).

The review identified various CSF shunt systems utilized in the cohort. VP shunts were the most prevalent type, explicitly documented in 28 patients (75.7%). Other specified systems included cystoperitoneal (CP) shunts (N=2, 5.4%), a lumboperitoneal (LP) shunt (N=1, 2.7%), and an arachnoid cyst shunt (N=1, 2.7%). We searched for the specific valve types, and our results show 17 adjustable types (45.95%), 18 non-adjustable types (48.65%), and two unreported. In our review, an anti-siphon device was present in only 11 patients (29.73%) (see Appendix A).

**Figure 5:**
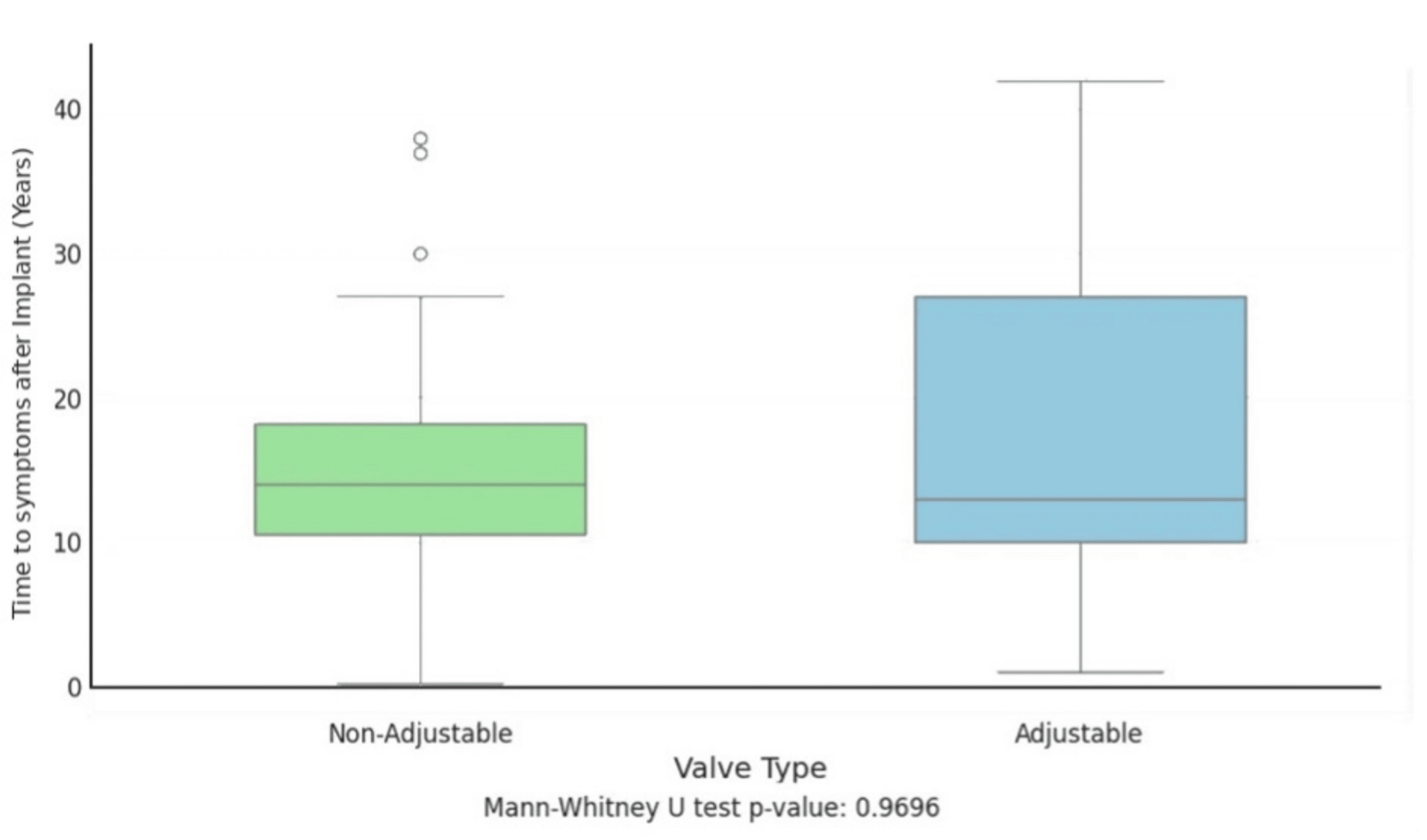
Comparison of time from last shunt implantation to symptom onset between patients with adjustable and non-adjustable valves. No significant difference was observed between groups using the Mann–Whitney U test (p = 0.9696).

The time interval from the last shunt procedure to the onset of neurological symptoms potentially related to overdrainage was assessed for 33 patients with quantifiable data. This latency period exhibited substantial variation, with a range spanning from 1.5 months (0.125 years) to 42 years. Reflecting the skewed nature of this distribution, the median time to symptom onset was 14 years. The interquartile range (IQR) was 17 years, with the first quartile (Q1) at 10 years and the third quartile (Q3) at 27 years, indicating that half of these patients developed symptoms between 10 and 27-years post-procedure. We didn’t find any statistical significance between the two valve types (p=0.9696) (Figure 5).

The duration of cervical signs was documented in 21 patients. Signs were present for less than three months in six patients (28.57%), between three and six months in one patient (4.76%), and between seven and 12 months in four patients (19.05%). A duration of one to two years was observed in two patients (9.52%). Longer durations were noted between two and five years in four patients (19.05%) and for more than five years in another four patients (19.05%).

Clinical presentations commonly involved symptoms and features noticed in cervical myelopathy, such as upper motor neuron (UMN) signs associated with motor paresis. Gait disturbance or ataxia was highly prevalent. Sensory disturbances were also common, often accompanied by hand and fine motor difficulties such as clumsiness and impaired dexterity. Neck pain and headache were reported less frequently (see Appendix B).

The pathognomonic and consistent reported radiological feature was cervical epidural venous plexus enlargement or engorgement (n=34) with cord compression. Other frequent findings included diffuse pachymeningeal or dural enhancement (n=15), evidence of spinal cord compression (n=10), often attributed to the venous engorgement, and slit or markedly small ventricles (n=10). Associated abnormalities comprised signs of myelopathy or myelomalacia, such as intramedullary T2 signal changes (n=8), and spinal cord deformity (n=5). Less commonly observed were dural thickening (n=4) and pituitary gland enlargement (n=4). Treatment strategies varied, with 12 cases (32.4%) involving combined approaches rather than single interventions. A significant trend involved shunt valve management, specifically converting non-adjustable (21 pre-intervention vs. 4 post-intervention) to adjustable valves (14 pre-intervention vs. 29 post-intervention), reflecting a strong shift towards adjustable systems. Anti-siphon devices were added in nine interventions, and notably, all patients with such devices (pre-existing or added) showed improvement (100%), compared to 90.9% improvement in those without. Intervention timing correlated with outcomes; early intervention (symptoms ≤1 year) yielded a 100% success rate, while delayed intervention (>1 year) achieved 92.9% success (see Appendix C). 

Discussion

Pathophysiology

The pathophysiology underlying Miyazaki syndrome is thought to involve primarily two classical models: the Monro-Kellie doctrine and the Starling resistor hypothesis [2,14-16]. The proposed pathomechanism of Miyazaki syndrome was described in detail by Barami and Barami & Sood [38,39]. The anatomy of the cranial venous outflow tracts is crucial in the pathophysiology of cerebral venous overdrainage. When a person is lying supine, venous return from the brain occurs predominantly through the internal jugular veins (IJVs). However, on assuming an upright posture, the IJVs may partially collapse under atmospheric pressure, causing venous blood to be diverted through alternative outflow routes. In this setting, drainage is increasingly shifted toward non-jugular pathways, particularly the vertebral venous plexus, which is less prone to collapse [24]. Chronic intracranial hypotension thus preferentially directs increased venous flow into the cervical epidural veins, particularly when typical cranial venous outflow pathways, such as the IJVs, become compromised or narrowed due to anatomical or positional compression ("styloidogenic jugular compression") [22,34].

As it is known, the Monro-Kellie doctrine states that within the fixed-volume intracranial-spinal space, a reduction in one component necessitates compensatory expansion of another. In the case of Miyazaki syndrome, the CSF volume decrease will be compensated by expansion of the venous system [14,24]. In the early phase of CSF hypovolemia, the compliant venous compartment may buffer the reduction in CSF volume by expanding the dural venous sinuses, producing pachymeningeal enhancement with vascular engorgement, and enlarging the spinal epidural venous plexus. When this compensatory venous response is insufficient, leakage of fluid from distended pachymeningeal veins may occur [2].

**Figure 6:**
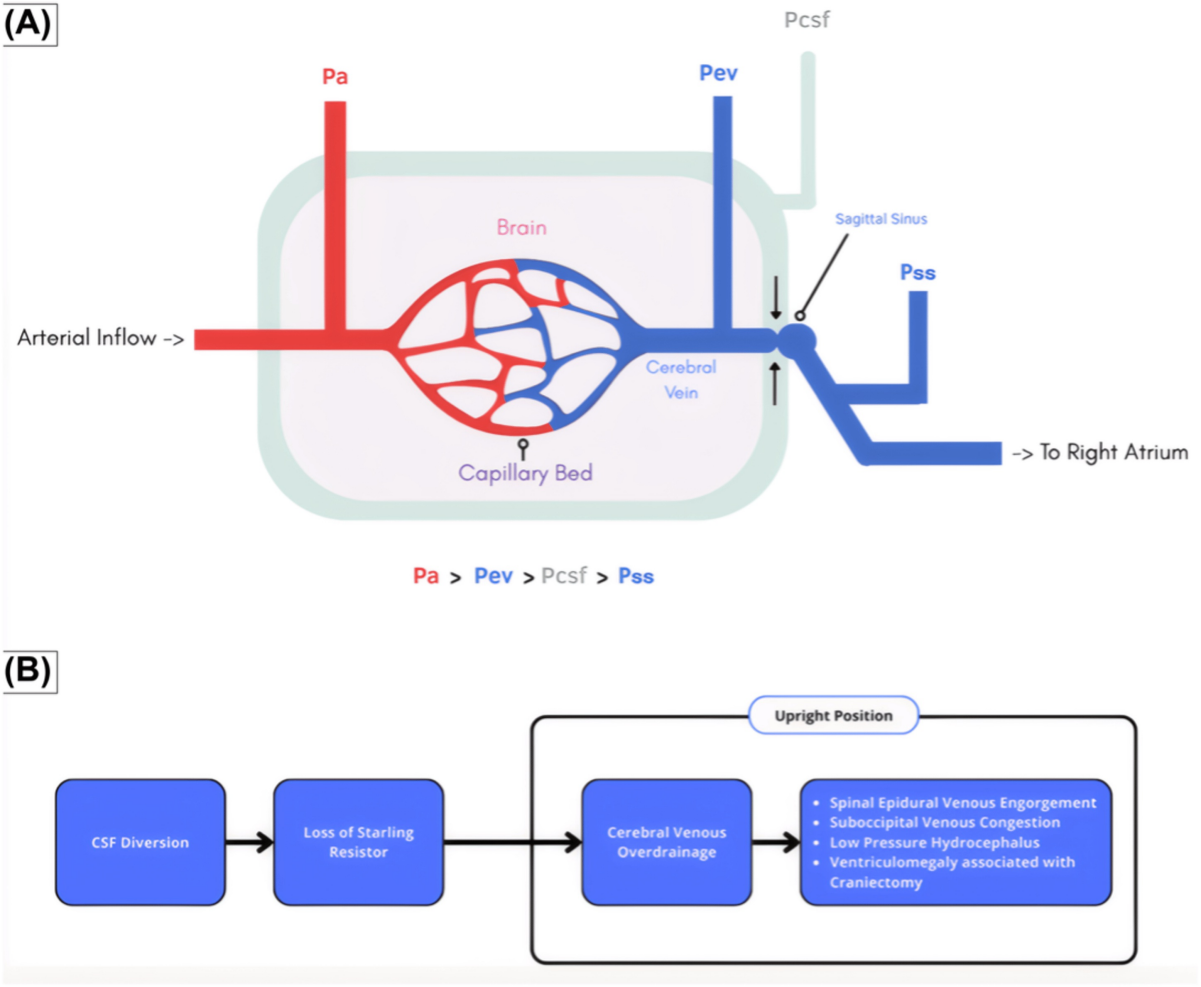
Intracranial hydrostatic pressure relationships and mechanism of cerebral venous overdrainage following CSF diversion. Under physiological conditions, a descending pressure gradient—Pa > Pev > Pcsf > Pss—maintains the Starling resistor effect, which controls cerebral venous outflow and supports CSF absorption. When CSF is excessively diverted, this pressure hierarchy is disrupted, leading to collapse of the venous outflow tract and cerebral venous overdrainage. In the upright posture, this results in a spectrum of findings including epidural venous engorgement, venous congestion in the posterior fossa, low-pressure hydrocephalus, and ventriculomegaly, hallmarks of Miyazaki syndrome. Pa, arterial pressure; Pev, cerebral venous pressure (pressure within the cerebral venous outflow tract); Pcsf, cerebrospinal fluid pressure; Pss, superior sagittal sinus pressure; CSF, cerebrospinal fluid. The image was created using Canva (Canva, Pty Ltd., Sydney, Australia).

The Starling resistor model proposes that intracranial fluid compartments are organized along a descending hydrostatic pressure gradient, extending from the arterial circulation toward the venous outflow system [14]. The Starling resistor mechanism helps preserve steady flow through collapsible vascular channels, including the bridging veins that traverse the subarachnoid CSF space [2]. CSF pressure and cerebral venous pressure are physiologically interdependent, and this interaction is partly governed by the Starling resistor effect. This mechanism helps maintain flow through compliant venous channels that are susceptible to collapse, including the bridging veins coursing through the subarachnoid CSF space [40]. This mechanism preserves the normal intracranial hydrostatic pressure gradient, in which arterial pressure exceeds venous pressure, followed by CSF pressure and then superior sagittal sinus pressure (Parterial > Pvenous > PCSF > PSSS) (Figure 6).

During transition to the upright position, superior sagittal sinus pressure normally decreases; in the absence of an effective compensatory mechanism, this pressure drop could promote excessive venous drainage and siphoning, ultimately contributing to intracranial hypotension [2]. Under normal conditions, this mechanism applies gentle external pressure at the bridging vein junctions, helping to prevent excessive venous outflow while maintaining venous pressure above CSF pressure and preserving forward flow before the compression point. When CSF is diverted, as occurs with VP shunting, loss of CSF volume may weaken this Starling resistor effect. As a result, the reduced CSF compartment can no longer provide enough pressure to support controlled compression of the bridging vein junctions, allowing cerebral venous overdrainage to develop. This process may be amplified in the upright position, when partial collapse of the IJVs can redirect venous blood through alternative collateral pathways. In this context, cerebral venous outflow may be shifted toward the spinal epidural venous plexus through its communication with the intracranial venous system via the suboccipital sinus. Over time, this redirected flow may cause enlargement and engorgement of the epidural venous plexus, which can exert mass effect on the spinal cord or exiting nerve roots [2]. Based on the review by Huang et al. (2022), the function of artificial Anti-Siphon Devices (ASDs) shares a conceptual similarity with the physiological Starling resistor mechanism, particularly as exemplified by the IJV collapse in the upright posture, which the authors explicitly refer to as a "natural ASD" [40]. Both mechanisms serve to counteract excessive fluid drainage from the cranium driven by hydrostatic pressure changes when an individual moves from a supine to an upright position. The Starling resistor achieves this for venous blood by utilizing the surrounding CSF pressure to induce a partial collapse (and thus increased resistance) in bridging veins or the IJV as downstream venous pressure drops due to gravity. Similarly, ASDs are designed to increase resistance within the CSF shunt pathway, specifically when the siphon effect (a consequence of hydrostatic pressure) becomes significant in the upright position, thereby mimicking the natural regulatory function to prevent overdrainage, albeit through various artificial mechanical means (diaphragm, gravitational, or flow-regulation) [40].

Clinical and Radiologic Findings

Miyazaki syndrome is defined by progressive compressive myelopathy symptoms in the presence of radiologic intracranial hypotension, with or without overdrainage symptoms. Our literature review confirmed this with (UMN) signs (spasticity, hyperreflexia, Babinski), sensory disturbances, and fine motor difficulties as characteristic features; motor deficits (most frequently spastic tetraparesis/quadriparesis), and highly prevalent gait disturbance/ataxia. The two cases presented illustrate this syndrome and underscore the clinical distinctions found in the literature. As illustrated by our cases, chronic intracranial hypotension can present with subdued or even absent clinical signs of overdrainage.

While brain MRI shows idiopathic intracranial hypertension (IIH) signs, the defining radiological feature of Miyazaki syndrome, consistently found in our review (n=34), is marked cervical epidural venous plexus engorgement on spinal MRI. This engorgement often causes secondary cord compression (n=10), deformation (n=5), or myelopathic changes (n=8), which are less relevant in pure shunt-induced Miyazaki syndrome [6,24,34,41,42].

Treatment Modalities

The goal of treatment in Miyazaki syndrome is to prevent CSF overdrainage. Surgical decision-making is highly individualized, depending on the patient's presenting symptoms, the severity of overdrainage, the degree of venous congestion seen on imaging, the type of valve already in place, and whether an anti-siphon device is present. Current shunt valve systems offer several options to reduce excessive CSF drainage. These include high-pressure valves, flow-controlled valves, adjustable (programmable) pressure valves, and distal catheters with a smaller internal diameter. Anti-siphon devices provide a further means of counteracting overdrainage, particularly in the upright posture; these include both fixed and adjustable gravitational units, which may be used alone or in combination with adjustable pressure valves [43]. In a retrospective 10-year cohort of 159 adult shunt patients, Mpakopoulou et al. (2012) reported that programmable-pressure valves halved overdrainage-related morbidity: subdural fluid collections occurred in only 4% of the programmable-valve group versus 17.5% in the fixed-pressure cohort, and the cumulative shunt revision rate was reduced from 40.0% to 20.1% [44]. Both Ros et al. (2021) and our review results underscore that adjustable-pressure valves permit noninvasive titration of outflow resistance and serve as the logical first step once overdrainage is recognized [43]. In our 37 patients, 78% underwent valve reprogramming or conversion to a programmable device, mirroring the emphasis on pressure modulation. By contrast, in our review, valve adjustability did not significantly influence the timing of symptomatic myelopathy. Among the 33 patients with quantifiable latency data, the median interval from the last shunt procedure to symptom onset was 14 years, and this did not differ significantly between adjustable and non-adjustable valves (Mann-Whitney U, p = 0.9696). Although adjustable valves are theorized to reduce overdrainage, they were not associated with a longer symptom latency in our series. This result should be interpreted with caution, given the small sample size and the heterogeneity of the pooled case reports, which limit statistical power. Clinically, it suggests that valve adjustability alone should not be relied upon to prevent the delayed venous overdrainage underlying Miyazaki syndrome, and that anti-siphon measures and vigilant follow-up remain important even when an adjustable valve is in place. Larger, prospectively collected cohorts will be essential to determine whether valve type truly affects the incidence or onset of the syndrome. ASDs are in-line resistance mechanisms that counteract posture-dependent CSF “siphoning” by introducing a threshold pressure drop once the hydrostatic column exceeds a set point. In their integrative model, Ros et al. (2021) identify initial siphoning as the trigger for ventricular collapse, CSF isolation, and secondary venous hypertension, and therefore advocate primary prophylaxis with ASDs (or gravitational valves) at the time of shunt implantation or revision to blunt the postural intracranial pressure drop and interrupt the overdrainage cascade [43]. In the observed series of 37 adults with Miyazaki syndrome, ASDs were added in nine cases, each of which demonstrated rapid symptom relief and MRI-confirmed resolution of dorsal column edema, yielding a 100% response rate. Given their proven efficacy, prompt ASD placement, ideally at the first sign of overdrainage or even prophylactically in patients at high risk for siphoning, should accompany valve pressure adjustment. Careful selection of the ASD opening threshold is essential to avoid excessive resistance and underdrainage in the supine position [40,43]. Beyond these direct shunt system modifications, Ko et al. (2020) recently described ETV as a successful intervention for OSAM in a patient with coexisting obstructive hydrocephalus [19]. This case suggests ETV may address not only the primary pathology of hydrocephalus but also potentially mitigate the venous congestion integral to OSAM. However, this remains, to our knowledge, the sole published instance of ETV employed specifically for OSAM. Consequently, while the literature on OSAM is expanding, data supporting ETV in this specific context are currently limited [19]. 

Crucially, direct spinal decompression surgery should be avoided in all instances of epidural venous plexus engorgement. This is due to the substantial risk of epidural bleeding from the engorged veins and inferior results, as it does not tackle the primary pathology.

In essence, managing Miyazaki syndrome revolves around correcting CSF overdrainage. The primary surgical step usually involves adjusting an existing programmable valve. If a fixed-pressure valve is in place, it's typically revised to a programmable one for more precise pressure management. Adding an anti-siphon device should be a key consideration to counteract siphoning. 

Limitations

Several limitations may influence the interpretation of this case series and systematic review. Primarily, the limited documented literature on Miyazaki syndrome necessitated a focus on case reports and case series. While these studies offer valuable insights into individual presentations and outcomes, their inherent limitations, such as potential reporting and selection biases, must be considered. The reliance on data extracted exclusively from these study designs introduces a predisposition to bias that might not be present in larger studies. Furthermore, the heterogeneity in reporting across the included studies posed challenges in conducting more comprehensive quantitative analyses. Finally, the overall sample size of 37 patients, derived from the aggregated case reports and series, is relatively small, which inherently limits the generalizability of the conclusions drawn from this review to the broader population.

## Conclusions

Miyazaki syndrome is a rare but reversible cause of progressive cervical myelopathy in shunted patients. As our two cases and systematic review demonstrate, early recognition of intracranial hypotension and prompt correction of CSF overdrainage, particularly through anti-siphon device placement and valve optimization, can reverse venous engorgement and prevent permanent neurological injury. In refractory cases, shunt ligation or endoscopic third ventriculostomy offers definitive relief. Maintaining a high index of suspicion in any shunted patient with progressive myelopathy and initiating a prompt, stepwise treatment plan gives patients the best chance of recovery. Larger, prospectively collected cohorts are nonetheless needed to clarify the pathophysiology and refine the treatment algorithms.

## Appendices

Appendix A 

**Table 1:** Summary of patient characteristics, shunt configurations, and presence of anti-siphon devices in published cases of overshunting-associated myelopathy. This table presents individual patient data extracted from included case reports and case series, including age, gender, indication for shunt placement, shunt type, valve type (adjustable vs. non-adjustable), and presence of anti-siphon devices. VP, ventriculoperitoneal; LP, lumboperitoneal; CP, cystoperitoneal; ETV, endoscopic third ventriculostomy; CSF, cerebrospinal fluid; N/A, not available.

Study	Age/gender	Indication of shunt implantation	Shunt type	Valve type	Anti-siphon device
Wolfe et al. (2207) [24]	17/M	Pilocytic astrocytoma (posterior cavity)	VP shunt; no antisiphon device (ASD)	Non-adjustable	No
Ulrich et al. (2013) [5]	17/M	Obstructive hydrocephalus caused by retrocerebellar arachnoid cyst	VP shunt, replaced 6 years later (shunt failure)	Non-adjustable	Yes
Howard et al. (2014) [7]	26/F	Congenital hydrocephalus	VP shunt with 2 revisions, medium pressure	Non-adjustable	No
Ko et al. (2020) [19]	67/F	Obstructive hydrocephalus (aqueductal stenosis)	First valve: VP shunt constant pressure; Revision: Endoscopic third ventriculostomy (ETV) + programmable valve (>180 mmH₂O)	Adjustable	No
Caruso et al. (2015) [15]	35/M	Supra- and infratentorial arachnoid cysts, agenesis of the corpus callosum	VP shunt, high-pressure valve; ASD not used	Non-adjustable	No
Ho et al. (2016) [33]	64/F	SAH-induced hydrocephalus	VP shunt, medium pressure	Non-adjustable	No
Ho et al. (2016) [33]	22/F	Congenital hydrocephalus caused by posterior fossa arachnoid cyst	VP shunt (shunt revision at age 17)	Non-adjustable	Yes
Miyazaki et al. (1998) [1]	53/M	SAH-induced hydrocephalus	Flow-regulated valve; low pressure; no ASD	Non-adjustable	No
Matsumoto et al. (2002) [26]	67/M	Obstructive hydrocephalus caused by pilocytic astrocytoma	Programmable valve system	Adjustable	No
Wingerchuk et al. (2005) [21]	72/F	Posterior fossa meningioma	No information	N/A	No
Liu et al. (2006) [25]	18/F	Large porencephalic	Distal slit-valve system	Non-adjustable	No
Cardoso et al. (2014) [27]	32/M	Communicating hydrocephalus	VP shunt (first non-programmable, then programmable)	Adjustable	No
Dantas et al. (2016) [28]	33/F	Communicating hydrocephalus	VP shunt, high-pressure valve	Non-adjustable	No
Martínez-Lage et al. (2009) [10]	69/M	Hydrocephalus after cerebellar cyst resection	VP shunt	Non-adjustable	No
Amano et al. (2018) [3]	77/M	SAH-induced hydrocephalus	VP shunt; no ASD	Non-adjustable	No
Kovács et al. (2018) [14]	33/F	Communicating hydrocephalus	pressure-adjustable VP Hakim valve system	Adjustable	Yes
van Lanen et al. (2024) [4]	41/F	Hydrocephalus due to IVH	VP shunt	Adjustable	Yes
Koge et al. (2018) [20]	50/F	Hydrocephalus due to IVH	VP shunt	Non-adjustable	No
Adib et al. (2018) [8]	45/M	Congenital hydrocephalus	VP shunt	Adjustable	Yes
Wernz et al. (2024) [22]	36/M	Idiopathic hydrocephalus	VP shunt	Adjustable	No
Hara et al. (2020) [34]	38/M	Retrocerebellar arachnoid cyst	Cysto-peritoneal shunt	Adjustable	Yes
Hara et al. (2020) [34]	83/F	NPH after SAH and coiling	VP shunt	Adjustable	Yes
Lee & Park (2023) [23]	55/M	Hydrocephalus following spontaneous SAH	VP shunt	Adjustable	No
Fujimori et al. (2021) [29]	52/M	Hydrocephalus after traumatic SAH (due to a traffic accident)	VP shunt	Non-adjustable	No
Sato et al. (2018) [30]	46/M	Idiopathic hydrocephalus	VP shunt	Adjustable	No
Fredrickson et al. (2018) [31]	29/F	Communicating hydrocephalus	LP shunt	Non-adjustable	Yes
Valgaeren et al. (2022) [32]	47/F	Cerebral arachnoid cyst	Arachnoid cyst shunt	N/A	No
Vister et al. (2021) [17]	75/M	Obstructive hydrocephalus colloid cyst	First valve: Unishunt (10-15 cm H₂O) Revision: Miethke gravity-assisted (10/40 cm H₂O)	Adjustable	Yes
Ueno et al. (2023) [18]	37/F	Congenital Hydrocephalus (6mo old)	VP shunt	Non-adjustable	No
Tavares et al. (2025) [6]	33/F	Hydrocephalus secondary to congenital cranial deformity with stenosis of the Sylvian aqueduct (diagnosed in early infancy)	First valve: Initially, a right VP shunt placed in infancy; Revision: A second left VP shunt was implanted; eventually, the left shunt valve was replaced with a CODMAN CERTAS plus programmable valve (set at 18 cm H₂O)	Adjustable	No
Ichikawa et al. (2021) [9]	42/M	Arachnoid cyst (AC) in the left frontal convexity causing papilledema and bilateral chronic subdural hematoma;	First valve: Initially a CP shunt with a nonprogrammable valve; Revision: Hakim programmable valve (Codman and Shurtleff Inc., Raynham, Massachusetts, USA) combined with an anti-siphon device (SiphonGuard, Codman, and Shurtleff Inc.)	Adjustable	Yes
Park & Park (2021) [16]	55/M	NPH following aneurysmal SAH (post-craniotomy and aneurysm clipping)	VP shunt	Non-adjustable	No
Várallyay et al. (2019) [2]	38/F	Infant hydrocephalus	VP shunt	Adjustable	No
Várallyay et al. (2019) [2]	37/F	Hydrocephalus due to aqueductal stenosis	VP shunt	Non-Adjustable	Yes
Várallyay et al. (2019) [2]	42/M	Hydrocephalus due to aqueductal stenosis	VA shunt reversed to VP shunt	Adjustable	No
Várallyay et al. (2019) [2]	26/F	Hydrocephalus due to aqueductal stenosis	VP shunt (endoscopic 3rd ventriculotomy)	Adjustable	No
Várallyay et al. (2019) [2]	16/M	Hydrocephalus due to meningitis	VP shunt	Non-adjustable	No

Appendix B 

**Table 2:** Summary of clinical presentation in patients with overshunting-associated myelopathy. This table details the time interval between the last shunt implantation and symptom onset, duration of cervical signs, and documented neurological findings for each included case. Clinical-sign codes: SENS, sensory disturbance; MOT, motor weakness/paralysis; TONE, spasticity and reflex hyperexcitability; COORD, coordination and gait dysfunction; PAIN, headache/axial/radicular pain; AUTO, autonomic dysfunction; CN/VIS, cranial nerve or visual/sensory deficits; COG, cognitive or neuropsychological changes; OTH, miscellaneous/systemic findings.

Study	Symptom onset after most recent shunt procedure	Duration of cervical symptoms	Clinical presentation								
			MOT	SENS	TONE	COORD	PAIN	CN/VIS	AUTO	COG	OTH
Miyazaki et al. (1998) [1]	1.25 years	7 months	−	+	+	+	−	−	−	−	−
Matsumoto et al. (2002) [26]	2 years	N/A	−	−	+	+	+	−	−	−	−
Wingerchuk et al. (2005) [21]	23 years	4 years	+	+	+	−	−	−	−	−	−
Liu et al. (2006) [25]	16 years	4 months	+	−	+	−	−	−	−	−	−
Wolfe et al. (2007) [24]	15 years	Unknown	+	−	+	−	−	−	−	−	−
Ulrich et al. (2013) [5]	14 years	2 years	+	−	−	−	−	−	−	−	−
Howard et al. (2014) [7]	12 years	4 weeks	−	+	+	+	−	−	−	−	−
Cardoso et al. (2014) [27]	10 years	Few months	+	−	+	−	−	−	−	−	−
Ko et al. (2020) [19]	42 years	Unknown	−	+	−	+	+	−	−	−	−
Caruso et al. (2015) [15]	19 years	7 years	+	−	+	−	−	−	−	−	−
Dantas et al. (2016) [28]	2 years	N/A	+	+	−	−	+	+	−	−	−
Ho et al. (2016) [33]	14 years	12 months	+	+	+	+	−	−	−	−	−
Ho et al. (2016) [33]	5 years	2 months	+	−	+	−	+	−	−	−	+
Martínez-Lage et al. (2009 [10]	38 years	Few years	−	−	+	+	−	−	−	+	−
Amano et al. (2013) [3]	10 years	3 years	+	+	+	−	−	−	−	−	−
Kovács et al. (2018) [14]	10 years	A few months	−	+	−	−	−	−	−	−	−
van Lanen et al. (2024) [4]	Several months	Unknown	+	−	+	−	−	−	−	−	−
Koge et al. (2018) [20]	16 years	1 month	+	+	+	−	−	−	−	−	−
Adib et al. (2018) [8]	8 years	Progressive over 8 years, with severe deterioration in the last 5 months	+	−	+	+	−	−	−	−	−
Wernz et al. (2024) [22]	12 years	1 year	−	+	+	+	−	−	−	−	−
Hara et al. (2020) [34]	31 years	1 year	+	+	+	+	+	−	−	−	−
Hara et al. (2020) [34]	11 years	2 years	+	+	+	+	−	−	−	−	−
Lee & Park (2023) [23]	15 years	1 month	+	+	−	+	−	−	−	−	+
Fujimori et al. (2021) [29]	27 years	3 years	−	+	−	+	−	−	+	−	−
Sato et al. (2018) [30]	14 years	Progressive over several years	+	+	+	−	−	+	−	−	−
Fredrickson et al. (2018) [31]	1.5 months	1.5 months	−	−	−	−	+	−	−	−	−
Valgaeren et al. (2022) [32]	N/A	Slowly progressive	−	−	−	−	+	−	−	−	−
Vister et al. (2021) [17]	27 years	N/A	−	+	+	+	−	−	−	−	−
Ueno et al. (2023) [18]	37 years	3 years	+	−	+	+	−	−	−	−	−
Tavares et al. (2025) [6]	N/A	10 years	+	−	+	+	−	−	+	−	−
Ichikawa et al. (2021) [9]	32 years	12 years	+	−	+	+	−	−	+	−	−
Park & Park (2023) [16]	14 years	2 months	+	+	+	+	+	−	−	−	−
Várallyay et al. (2019) [2]	1 year	N/A	−	+	−	+	−	+	−	−	−
Várallyay et al. (2019) [2]	30 years	N/A	−	−	−	+	−	+	−	−	+
Várallyay et al. 2019 [2]	27 years	N/A	+	−	−	+	−	−	−	−	−
Várallyay et al. (2019) [2]	Several years	N/A	−	−	+	+	+	+	−	−	+
Várallyay et al. (2019) [2]	14 years	N/A	+	+	−	−	−	−	−	−	−

Appendix C 

**Table 3:** Radiological features, treatment strategies, and clinical outcomes in patients with overshunting-associated myelopathy. The table summarizes neuroradiological findings, interventions performed, and reported clinical outcomes across all included cases. Radiology codes: MENH, pachy- or dural meningeal enhancement/thickening; EPVP, epidural or vertebral venous plexus enlargement/engorgement; VENT, slit or markedly reduced ventricles; SUBD, subdural hygroma/fluid collection; VEN, venous-sinus or jugular-vein distension/collapse; PIT, pituitary-gland enlargement or convexity; HERNI, tonsillar herniation or brainstem sagging/low-lying cerebellum; SHIFT, brain or midline shift; BONE, calvarial/skull thickening; CORD, spinal cord compression/deformation/T2 signal change/myelopathy; EXTRA, extradural/epidural mass or hematoma; STEN, spinal canal stenosis or nerve root compression. Treatment codes: CLOSE, shunt closure, ligation, or transection; REMOVE, complete shunt removal; NONE, no surgical treatment/observation; REPL, shunt or valve replacement/revision (including programmable systems); ADJ, valve pressure adjustment; GRAV, addition of anti-siphon or gravitational device; DECOMP, spinal decompression, laminectomy, or hematoma evacuation; FUSE, spinal fusion/instrumentation; ETV, endoscopic third ventriculostomy; MED, medical management (e.g., corticosteroids); REHAB, rehabilitation or physiotherapy.

Study/age/gender	Radiologic findings	Treatment	Outcome
Miyazaki et al. (1998) [1]	SHIFT, EPVP, MENH	CLOSE	Improved
Matsumoto et al. (2002) [26]	MENH, EXTRA	REMOVE	Improved
Wingerchuk et al. (2005) [21]	VENT, MENH, SUBD, EPVP	NONE	Worsening
Liu et al. (2006) [25]	EPVP	REPL	Improved
Wolfe et al. (2007) [24]	MENH, SHIFT, VEN, EPVP	REPL	Improved
Ulrich et al. (2013) [5]	BONE, PIT, EPVP	REPL	Slight Improvement
Howard et al. (2014) [7]	MENH, PIT, HERNI, EPVP	REPL	Improved
Cardoso et al. (2014) [27]	EPVP	ADJ	Improved
Caruso et al. (2015) [15]	EPVP	CLOSE	Improved
Dantas et al. (2016) [28]	EPVP	REPL	Improved
Ho et al. (2016) [33]	MENH, EPVP	REPL	Improved
Ho et al. (2016) [33]	MENH, VENT, EPVP	REPL	Improved
Martínez-Lage et al. (2009) [10]	MENH, PIT, EPVP	REPL	Improved
Amano et al. (2018) [3]	EPVP	ADJ, DECOMP	Improved
Kovács et al. (2018) [14]	MENH, VEN, PIT, EPVP, CORD	ADJ, REPL, CLOSE, GRAV	Improved
van Lanen et al. (2024) [4]	VENT, CORD, EPVP	REPL	Improved
Koge et al. (2018) [20]	CORD, EPVP, MENH, VENT	REPL	Improved
Adib et al. (2018) [8]	VENT, MENH, EPVP, CORD	DECOMP, REPL, ADJ	Improved
Wernz et al. (2024) [22]	VENT, SUBD, EPVP, CORD, HERNI, VEN	ADJ	Slight improvement
Hara et al. (2020) [34]	EPVP, CORD	REPL, GRAV	Improved
Hara et al. (2020) [34]	EXTRA, EPVP, CORD	GRAV	Improved
Lee & Park (2023) [23]	VENT, EXTRA, EPVP, CORD	DECOMP, REPL, ADJ	Improved
Fujimori et al. (2021) [29]	CORD, EPVP, STEN, MENH	CLOSE	Improved
Sato et al. (2018) [30]	VENT, EPVP, CORD, VEN	REPL, ADJ	No improvement
Fredrickson et al. (2018) [31]	VENT, EPVP, STEN	FUSE	Improved
Valgaeren et al. (2022) [32]	MENH, VEN, EPVP, CORD	ADJ	Improved
Vister et al. (2021) [17]	VENT, SUBD, EPVP, STEN, CORD	CLOSE, REPL, GRAV	1st - Worsening Last - Improvement
Ueno et al. (2023) [18]	MENH, BONE, EPVP, CORD	CLOSE	Improved
Ko et al. (2020) [19]	STEN, EPVP, CORD	DECOMP, ETV, REPL, ADJ	1st: Worsening Last: Improvement
Tavares et al. (2025) [6]	EXTRA, CORD, EPVP	CLOSE, REPL	Improved
Ichikawa et al. (2021) [9]	EPVP, HERNI, MENH	REPL, GRAV, ADJ	Improved
Park & Park (2023)[16]	EXTRA, CORD, STEN	DECOMP, REPL, ADJ, MED, FUSE, REHAB	Improved
Várallyay et al. (2019) [2]	EPVP, CORD	ADJ	Improved
Várallyay et al. (2019) [2]	EPVP, CORD	REPL	Improved
Várallyay et al. (2019) [2]	EPVP, STEN	REPL, ADJ	Improved
Várallyay et al. (2019) [2]	EPVP, CORD	ADJ	Improved
Várallyay et al. (2019) [2]	EPVP, STEN	REHAB, REPL	Improved
